# The MORPHEUS II protein crystallization screen

**DOI:** 10.1107/S2053230X1500967X

**Published:** 2015-06-27

**Authors:** Fabrice Gorrec

**Affiliations:** a MRC Laboratory of Molecular Biology, Francis Crick Avenue, Cambridge Biomedical Campus, Cambridge CB2 0QH, England

**Keywords:** macromolecular crystallography, protein crystallization, crystallization screening, crystallization additives, heavy-atom derivatization, MORPHEUS II

## Abstract

High-quality macromolecular crystals are a prerequisite for the process of protein structure determination by X-ray diffraction. Unfortunately, the relative yield of diffraction-quality crystals from crystallization experiments is often very low. In this context, innovative crystallization screen formulations are continuously being developed. In the past, MORPHEUS, a screen in which each condition integrates a mix of additives selected from the Protein Data Bank, a cryoprotectant and a buffer system, was developed. Here, MORPHEUS II, a follow-up to the original 96-condition initial screen, is described. Reagents were selected to yield crystals when none might be observed in traditional initial screens. Besides, the screen includes heavy atoms for experimental phasing and small polyols to ensure the cryoprotection of crystals. The suitability of the resulting novel conditions is shown by the crystallization of a broad variety of protein samples and their efficiency is compared with commercially available conditions.

## Abstract 2

MORPHEUS II is a 96-condition initial crystallization screen formulated *de novo*. The screen incorporates reagents selected from the Protein Data Bank to yield crystals that are not observed in traditional conditions. In addition, the formulation facilitates the optimization and cryoprotection of crystals.

## 1. Introduction   

The technique of single-crystal X-ray diffraction enables the routine and precise structure determination of biological macromolecules at high resolution (Blow, 2002 ▸; Rupp, 2010 ▸). It has been applied extensively to proteins, DNA and RNA, with the PDB recently celebrating the amazing milestone of 100 000 deposited structures. This is essentially the result of a multitude of innovations and technological developments spanning the last few decades (Abola *et al.*, 2000 ▸; Fersht, 2008 ▸). Nonetheless, behind this success hides the struggle to produce purified samples, to obtain crystals with suitable diffraction quality and to subsequently reproduce/optimize them (Bergfors, 2009 ▸; Khurshid *et al.*, 2014 ▸).

The poor yield of suitable crystals can be explained by the concept called the ‘curse of dimensionality’: there are so many dimensions associated with the parameter space to be explored that it is problematic or impossible to perform an analysis that has any statistical significance. The underlying reason is that the combinations of reagents employed in crystallization trials alter the combinations of variables associated with the main parameters of crystallization (McPherson *et al.*, 1995 ▸); for example, the variables that are related to the nature of the protein and the experiment, the type of protein–protein interactions and so on. Hence, initial crystallization screening is a stochastic process (Carugo & Argos, 1997 ▸; Lomakin *et al.*, 1999 ▸) that usually requires various approaches and conditions.

A crystallization condition traditionally contains a precipitant, a buffer and an additive. There are now hundreds of well known crystallization reagents and the possible combinations used to formulate conditions in a systematic manner has grown to a very large number that cannot be captured in any practical way because the amount of sample and the screening technology are limiting (Carter & Carter, 1979 ▸; Gorrec, 2014 ▸). As a consequence, many laboratories have chosen an approach with a minimum number of conditions. A widespread minimal approach is to employ a set of conditions selected empirically to form a ‘sparse-matrix’ screen (Jancarik & Kim, 1991 ▸). In the last two decades, advances in automation and the increase in the number of crystal structures solved and deposited have stimulated the optimization of sparse-matrix screens, mostly in the form of sets of 96 conditions, as this is an automation-friendly format (Kimber *et al.*, 2003 ▸; Rupp & Wang, 2004 ▸; Newman *et al.*, 2005 ▸; Stock *et al.*, 2005 ▸; Newstead *et al.*, 2008 ▸; Fazio *et al.*, 2014 ▸).

Nevertheless, we have argued that the minimal approach may mean undersampling of conditions and therefore essential hits could be missed (Gorrec, 2013 ▸). Subsequently, novel formulations should still be investigated when possible. Besides, screens should evolve in parallel with the increasing complexity of the samples and the technical difficulties encountered during the process of structure determination. Notably, the demands of cryo-crystallography (Petsko, 1975 ▸; §[Sec sec3.1.1]3.1.1) as well as current and future solutions to the phase problem (Taylor, 2003 ▸; §[Sec sec3.1.2]3.1.2) should be taken into account.

Previously, we presented an innovative approach in a screen formulation called MORPHEUS (Gorrec, 2009 ▸). In order to reduce bias towards a subset of samples, the conditions were formulated *de novo* by integrating a larger number of reagents than traditionally employed. For this, novel mixes of reagents were investigated. Reagents that aided protein stabilization, crystallization and crystal cooling were included in the final formulation. The multiplexing of reagents has been performed previously for cryoprotectants (Garman & Mitchell, 1996 ▸), precipitants (Majeed *et al.*, 2003 ▸), buffer systems (Newman, 2004 ▸) and additives (McPherson & Cudney, 2006 ▸) (§[Sec sec3.1.3]3.1.3). The original MORPHEUS combined all of these innovations. It is worth highlighting two other particular design principles of MORPHEUS. Firstly, the inclusion of PDB-derived small molecules (as potential ligands) that were gathered into mixes of additives, sorted according to their chemical nature to avoid incompatibilities. Secondly, mixes of precipitants, additives and buffers were combined within a 96-condition three-dimensional grid screen using fixed ratios to facilitate easier screen preparation and follow-up optimizations.

Here, we present MORPHEUS II, a 96-condition protein crystallization screen formulated in continuity with previous work. MORPHEUS II follows the design principles of MORPHEUS; however, we introduced less common additives, for example metals that are amendable to the collection of anomalous data sets directly from screening conditions. We also included nondetergent sulfobetaines (NDSBs), polyamines, amino acids and monosaccharides, which are known to enhance the solubility and stability of many proteins. To complete the formulation of MORPHEUS II, four unusual ‘glycerol-like’ polyols have been added as cryoprotectants to aid flash-cooling. Finally, innovative buffer systems were included as part of the formulations. The suitability of the resulting conditions is shown by the crystallization of eight different protein samples and their efficiency is compared with commercially available conditions (§[Sec sec3.2]3.2).

## 2. Materials and methods   

### 2.1. Screen formulation   

The mixes of ligands, precipitants and buffers were combined using a fixed ratio of volumes for the stock solutions as employed in the original MORPHEUS screen: 0.5 stock precipitants + 0.1 stock additives + 0.1 stock buffer system + 0.3 water. Methods used to select the PDB-derived ligands, to design the screen and to prepare the stock solutions were also as described previously (Gorrec, 2009 ▸). Further details can be found in the Supporting Information concerning the four precipitant mixes (Supplementary Table S1) and the three buffer systems (Supplementary Table S2).

Although the additive-to-protein ratio preferably needs to be maximized (Danley, 2006 ▸), the concentrations of the mixes Divalent cations II, Akalis, Oxometalates and Lanthanides had to be lowered compared with other, more soluble and less reactive additives, such as monosaccharides and carboxylic acids. Relatively low concentrations (around 1 m*M*) are suitable for these particular additives according to others (Petsko, 1985 ▸; Trakhanov & Quiocho, 1995 ▸).

By empirical experimentation, stable and suitable combinations of reagents were found. Unfortunately, some traditional heavy atoms had to be excluded, such as those of the platinum group (chloride salts of platinum, osmium, iridium, ruthenium, rhodium and palladium) since they could not be solubilized and/or were unstable in solution. Of course, they can still be tested later on crystals already formed when necessary. The buffer system was removed from conditions B5–B8 to avoid precipitation (probably owing to formation of a chelate between a divalent cation and one of the corresponding buffers).

Also following an empirical approach, it was found that four small polyols that are not currently found in any commercially available screens vitrified samples during flash-cooling as efficiently as glycerol (*i.e.* typically 20–25% required to cryoprotect conditions): 1,2,4-butanetriol, 1,2,6-hexanetriol, 1,5-pentanediol and 1,1,1-tris(hydroxymethyl)propane. They were thus integrated into the precipitant mixes (Table 1 ▸ and Supplementary Table S1). X-ray diffraction tests with the mixes of polyols and polyethylene glycols (PEGs) flash-cooled in cryoloops were then used to adjust the concentration of cryoprotectants and ensured that the resulting diffraction patterns were free of background from ice. NDSBs are another group of reagents often used in sample preparation and crystallization additive screens. They have a relatively low frequency of occurrence in crystal structures, which suggests that their role may be less specific and therefore they have been integrated to the precipitant mixes.

### 2.2. Crystallization experiments   

The protein samples can be briefly described as follows: concanavalin A (‘Con’, molecular weight 27 kDa, concentration 13 mg ml^−1^, Sigma catalogue No. L7647 dissolved in 0.1 *M* Tris pH 8.5), polymerase III clamp–exonuclease complex (‘Pol’, 80 kDa, 10 mg ml^−1^; Rêgo *et al.*, 2013 ▸), ESCRT-II complex (‘E2H’, 115 kDa, 7 mg ml^−1^; Teo *et al.*, 2004 ▸), bar domain (‘Bar’, 6 mg ml^−1^, 29 kDa; Peter *et al.*, 2004 ▸), HIV capsid (‘HIV’, 25 kDa, 32 mg ml^−1^; Price *et al.*, 2014 ▸), coiled-coil domain of the cytosolic nucleic acid sensor LRRFIP1 (‘CCD’, 12 kDa, 9 mg ml^−1^; Nguyen & Modis, 2013 ▸), ubiquitin–protein ligase (‘UPL’, 21 kDa, 9 mg ml^−1^; Elliott *et al.*, 2014 ▸) and mRNA nuclear-export factor complex (‘NEF’, 68 kDa, 8 mg ml^−1^; Aibara *et al.*, 2015 ▸).

The original MORPHEUS screen was purchased from Molecular Dimensions Ltd (‘MORPHEUS I’, Lot No. 021-1-46) and the sparse-matrix screen ‘The JCSG+ Suite’ was purchased from Qiagen (Lot No. 54806713). Triplicate droplets of 300 nl final volume with a 2:1 protein-to-condition ratio were formed in MRC plates at 20°C using a Mosquito robot (TTP Labtech). Single droplets of 200 nl final volume (1:1 ratio) were prepared similarly for the ‘Bar’ sample only. The plates were swiftly sealed and centrifuged (1000 rev min^−1^, 1 min) and then kept at 18°C. Droplets were visualized with a stereo microscope after one week. Only obvious hits were taken into account (*i.e*. drops with crystals larger than 5 µm and with sharp edges). Details of the corresponding crystallization results can be found in the Supporting Information (Supplementary Table S3).

## 3. Results and discussion   

### 3.1. Formulation of MORPHEUS II   

The formulations of the 96 MORPHEUS II crystallization conditions are listed in Table 1 ▸. The 35 PDB-derived ligands selected to formulate MORPHEUS II can be found in Table 2 ▸. The recipes for preparing the eight additive mixes are listed in Table 3 ▸.

#### 3.1.1. Integration of cryoprotecting agents   

To obtain vitrification after flash-cooling, crystals are usually soaked briefly in a solution containing a cryoprotectant (most commonly glycerol). Many crystals are lost using this approach owing to the extensive handling and/or resulting variations in the composition of the mother liquor. A logical remedy for these issues is the use of crystallization conditions that are already cryoprotected. From this perspective, the amount of glycerol needed to successfully vitrify the conditions of Jancarik & Kim (1991 ▸) was determined by Garman & Mitchell (1996 ▸). Later, another study expanded these data with PEG 400, ethylene glycol and 1,2-propanediol (McFerrin & Snell, 2002 ▸). Adding the cryoprotectant directly to the formulations seems straightforward; however, the impact of introducing an additional reagent at high concentration on the yield of quality crystals was not investigated.

Cryoprotection should not bias formulations towards only a few cryoprotectants as main reagents since this would contribute to a further undersampling of initial screen conditions. We thought that more cryoprotectants should be tested as part of the development of novel conditions. Therefore, we integrated other polyols into the MORPHEUS II screen. Using polyols as cryoprotectants is beneficial since they are typically easy to handle and they display other interesting properties. For example, polyols are somewhat hygroscopic (Cohen *et al.*, 1993 ▸) and hence they also act as precipitants, altering both the hydration of proteins and the kinetics of vapour-diffusion experiments (Forsythe *et al.*, 2002 ▸; Collins, 2004 ▸). Finally, it should be pointed out that different cryoprotecting solutions cause different degrees of contraction upon flash-cooling and also affect cooling rates (Berejnov *et al.*, 2006 ▸; Alcorn & Juers, 2010 ▸). These parameters will affect the differential contraction between the macromolecular crystal and the mother liquor surrounding it, and hence the quality of the diffraction data obtained.

#### 3.1.2. Importance of additives   

Additives may alter the parameters of crystallization experiments in a myriad of ways. If used correctly, they can increase the chances of obtaining useful crystals. One approach is to test how reagents alter the stability and solubility of the sample prior to crystallization assays (Ericsson *et al.*, 2006 ▸; Izaac *et al.*, 2006 ▸). Also, binding to the protein may be investigated (Boggon & Shapiro, 2000 ▸). Nevertheless, other essential parameters are specific to crystallization. For example, it has been demonstrated that crystal growth can be altered by additives such as divalent metal cations (Trakhanov & Quiocho, 1995 ▸). Therefore, it is probably advisable to integrate as many additives as possible into our initial screen.

The positive impact of additives on the yield of crystals can be explained through the formation of new crystal contacts (Carugo & Djinović-Carugo, 2014 ▸). More complex molecules, for example polycarboxylic acids, sugars and polyamines, can bind to pockets in macromolecules and stabilize them or help them to adopt a particular conformation (Arakawa & Timasheff, 1982 ▸; Sauter *et al.*, 1999 ▸; Maclean *et al.*, 2002 ▸).

Polyamines were originally used with polynucleotides as they form favourable electrostatic interactions with DNA and RNA, leading to stable complexes (Bolton & Kearns, 1978 ▸; Drew & Dickerson, 1981 ▸). However, polyamines are now also regularly observed in complexes with proteins (*e.g.* spermine, PDB residue ID SPM, 105 occurrences in the PDB, Table 2 ▸). Zwitterionic organic chemicals, such as NDSBs (Table 2 ▸ and Supplementary Table S1) and also HEPES-like buffers (*e.g.* MES) can be used as additives/buffers for solubilization and may prevent aggregation or polymerization (Vuillard *et al.*, 1996 ▸). This may be explained through the abilities of molecules to shield specific apolar surface patches (Pusey *et al.*, 2007 ▸).

#### 3.1.3. Heavy atoms   

In order to solve a structure by SAD, some early work suggested that derivatization should be performed by soaking the crystals since crystal growth may be altered by heavy-atom binding and hence prevent lattice contacts or eventually produce a different crystal form (Petsko, 1985 ▸). However, the increasingly challenging nature of proteins studied with crystallography means that different crystal forms occur less frequently. In addition, it is well known that when heavy atoms are tested for derivatization by soaking crystals (Garman & Murray, 2003 ▸) the diffraction usually worsens or may even be lost (as for cryoprotectants; §[Sec sec3.1.1]3.1.1).

Integrating heavy atoms into the initial screen is therefore very desirable. This was demonstrated when crystals of the nitrogen regulation-related protein NreA grew in two similar MORPHEUS conditions but with two different mixes of additives. Subsequently, closely related crystal forms were obtained that contained either iodide (NreA–I; PDB entry 4iuh) or nitrate (NreA–NO_3_; PDB entry 4iuk) and enabled structure determination *ab initio* with experimental phasing (Niemann *et al.*, 2014 ▸). Although we cannot yet share similar results from MORPHEUS II, it is worth mentioning that we did not observe issues with diffuse scattering caused by conditions containing heavy atoms (Luft *et al.*, 2014 ▸), probably because of the very small proportion of the corresponding heavy atoms in the samples.

Finally, before thinking about derivatization, our goal was to increase our yield of initial (and novel) crystals. In this context, it is worth mentioning other work suggesting that multivalent metal ions such as yttrium can modulate protein–protein interactions and even mediate crystal contacts that help to form the crystal lattice: they specifically bind to acidic surface patches as well as bridging acidic side chains from neighbouring subunits (Zhang *et al.*, 2011 ▸).

#### 3.1.4. Integration of mixes of additives   

Use of mixes of additives during initial crystallization screening has been tested before with success (McPherson & Cudney, 2006 ▸). This strategy carries the risk that one component of a mix might have a deleterious effect and thereby mask the positive contribution of another. Nevertheless, if one of the additives from the mix participates in specific effects or interactions, the less specific addtive should be less pronounced. Also, by selecting components that have been regularly observed as ordered parts of crystal structures, the chances of incorporating molecules that play a positive role should be increased (Gorrec, 2009 ▸).

Finally, more than one type of additive may be required for crystal growth, as many structures contain multiple additives. This was demonstrated when the original MORPHEUS screen was used to crystallize the human endoplasmic reticulum aminopeptidase HERAP2 (PDB entry 3se6), where four additives and a buffer component are part of the crystal structure (Birtley *et al.*, 2012 ▸).

### 3.2. Crystallization experiments   

The overall yield of crystals was 11.3% with JCSG+, 17.2% with MORPHEUS and 16.5% with MORPHEUS II (Supplementary Table S3). Some may argue that our panel of test proteins may be more likely to crystallize under conditions containing high-molecular-weight PEG as the main precipitant (MORPHEUS screens; Page & Stevens, 2004 ▸). Different proteins may have specifically required relatively high salt concentrations and hence preferably crystallized in a sparse matrix (JCSG+). Others could also argue the most efficient pH range could have been anticipated (Kantardjieff & Rupp, 2004 ▸). Ultimately, the results will strongly depend on the subset of proteins tested.

The underlying problem is the numerous biases participating in the curse of dimensionality, notably those associated with the preparation of the screens/buffers (Wooh *et al.*, 2003 ▸), the type/number of protein samples selected (McPherson & Cudney, 2006 ▸) *etc*. In the end, no matter how sophisticated the statistical analysis and data mining of crystallization space, any of the approaches will only provide a basis for increasing the probability of crystallization success, but will never guarantee success for any particular protein (Rupp, 2003 ▸).

As a consequence, no strict conclusions should be drawn when comparing efficiency between screens. Surely, we have demonstrated the suitability of nontraditional and *de novo* formulated MORPHEUS II conditions for protein crystallization, at least for samples with a propensity to crystallize in conditions with high-molecular-weight PEG precipitant, while two other screens widely used by the protein crystallography community were used as controls. Nevertheless, we hope that MORPHEUS II will efficiently extend the range of available conditions and hence enable the crystallization of recalcitrant samples. A cryoprotected screen certainly reduces reproducibility issues observed during the cryoprotection of crystals.

## 4. Conclusions   

We have demonstrated that unusual and under-represented reagents can be combined to formulate suitable and useful conditions for protein crystal growth. The resulting screen is based on principles that have proven to be successful previously. The strategy of formulation reduces bias towards a subset of conditions or samples and integrates new mixes of reagents. MORPHEUS II has already increased our overall effectiveness with *de novo* structure determination enabled by including more heavy atoms in our initial screen.

## Supplementary Material

Crystallisation results with MORPHEUS II and other tables.. DOI: 10.1107/S2053230X1500967X/en5565sup1.pdf

## Figures and Tables

**Table 1:** Formulation of MORPHEUS II A mix of precipitants includes a high-molecular-weight PEG and a cryoprotectant (small polyol). Two precipitant mixes also include NDSBs. The formulations of the eight additive mixes can be found in Table 3 ▸. The formulations of the three buffer systems can be found in Supplementary Table S2. The roman numeral II was used to distinguish the new mixes from similarly named ones in the original MORPHEUS screen. AMPD, 2-amino-2-methyl-1,3-propanediol; BES, *N*,*N*-bis(2-hydroxyethyl)-2-aminoethanesulfonic acid; bis-tris, bis(2-hydroxyethyl)aminotris(hydroxymethyl)methane; GlyGly, glycylglycine; HEPES, 4-(2-hydroxyethyl)piperazine-1-ethanesulfonate; MES, 2-(*N*-morpholino)ethanesulfonic acid; MOPSO, 3-morpholino-2-hydroxypropanesulfonic acid; TEA, triethanolamine.

Well	Mix of precipitants	Mix of additives	Buffer system
A1	15%(*w*/*v*) PEG 3K, 20%(*v*/*v*) 1,2,4-butanetriol, 1%(*w*/*v*) NDSB 256	0.03*M* of each LiNaK	0.1*M* MOPSO/bis-tris pH 6.5
A2	12.5%(*w*/*v*) PEG 4K, 20%(*v*/*v*) 1,2,6-hexanetriol	0.03*M* of each LiNaK	0.1*M* MOPSO/bis-tris pH 6.5
A3	10%(*w*/*v*) PEG 8K, 20%(*v*/*v*) 1,5-pentanediol	0.03*M* of each LiNaK	0.1*M* MOPSO/bis-tris pH 6.5
A4	5%(*w*/*v*) PEG 20K, 25%(*w*/*v*) 1,1,1-tris(hydroxymethyl)propane, 1%(*w*/*v*) NDSB 195	0.03*M* of each LiNaK	0.1*M* MOPSO/bis-tris pH 6.5
A5	15%(*w*/*v*) PEG 3K, 20%(*v*/*v*) 1,2,4-butanetriol, 1%(*w*/*v*) NDSB 256	0.03*M* of each LiNaK	0.1*M* BES/TEA pH 7.5
A6	12.5%(*w*/*v*) PEG 4K, 20%(*v*/*v*) 1,2,6-hexanetriol	0.03*M* of each LiNaK	0.1*M* BES/TEA pH 7.5
A7	10%(*w*/*v*) PEG 8K, 20%(*v*/*v*) 1,5-pentanediol	0.03*M* of each LiNaK	0.1*M* BES/TEA pH 7.5
A8	5%(*w*/*v*) PEG 20K, 25%(*w*/*v*) 1,1,1-tris(hydroxymethyl)propane, 1%(*w*/*v*) NDSB 195	0.03*M* of each LiNaK	0.1*M* BES/TEA pH 7.5
A9	15%(*w*/*v*) PEG 3K, 20%(*v*/*v*) 1,2,4-butanetriol, 1%(*w*/*v*) NDSB 256	0.03*M* of each LiNaK	0.1*M* GlyGly/AMPD pH 8.5
A10	12.5%(*w*/*v*) PEG 4K, 20%(*v*/*v*) 1,2,6-hexanetriol	0.03*M* of each LiNaK	0.1*M* GlyGly/AMPD pH 8.5
A11	10%(*w*/*v*) PEG 8K, 20%(*v*/*v*) 1,5-pentanediol	0.03*M* of each LiNaK	0.1*M* GlyGly/AMPD pH 8.5
A12	5%(*w*/*v*) PEG 20K, 25%(*w*/*v*) 1,1,1-tris(hydroxymethyl)propane, 1%(*w*/*v*) NDSB 195	0.03*M* of each LiNaK	0.1*M* GlyGly/AMPD pH 8.5
B1	15%(*w*/*v*) PEG 3K, 20%(*v*/*v*) 1,2,4-butanetriol, 1%(*w*/*v*) NDSB 256	0.5m*M* of each Divalent cation II	0.1*M* MOPSO/bis-tris pH 6.5
B2	12.5%(*w*/*v*) PEG 4K, 20%(*v*/*v*) 1,2,6-hexanetriol	0.5m*M* of each Divalent cation II	0.1*M* MOPSO/bis-tris pH 6.5
B3	10%(*w*/*v*) PEG 8K, 20%(*v*/*v*) 1,5-pentanediol	0.5m*M* of each Divalent cation II	0.1*M* MOPSO/bis-tris pH 6.5
B4	5%(*w*/*v*) PEG 20K, 25%(*w*/*v*) 1,1,1-tris(hydroxymethyl)propane, 1%(*w*/*v*) NDSB 195	0.5m*M* of each Divalent cation II	0.1*M* MOPSO/bis-tris pH 6.5
B5	15%(*w*/*v*) PEG 3K, 20%(*v*/*v*) 1,2,4-butanetriol, 1%(*w*/*v*) NDSB 256	0.5m*M* of each Divalent cation II	
B6	12.5%(*w*/*v*) PEG 4K, 20%(*v*/*v*) 1,2,6-hexanetriol	0.5m*M* of each Divalent cation II	
B7	10%(*w*/*v*) PEG 8K, 20%(*v*/*v*) 1,5-pentanediol	0.5m*M* of each Divalent cation II	
B8	5%(*w*/*v*) PEG 20K, 25%(*w*/*v*) 1,1,1-tris(hydroxymethyl)propane, 1%(*w*/*v*) NDSB 195	0.5m*M* of each Divalent cation II	
B9	15%(*w*/*v*) PEG 3K, 20%(*v*/*v*) 1,2,4-butanetriol, 1%(*w*/*v*) NDSB 256	0.5m*M* of each Divalent cation II	0.1*M* GlyGly/AMPD pH 8.5
B10	12.5%(*w*/*v*) PEG 4K, 20%(*v*/*v*) 1,2,6-hexanetriol	0.5m*M* of each Divalent cation II	0.1*M* GlyGly/AMPD pH 8.5
B11	10%(*w*/*v*) PEG 8K, 20%(*v*/*v*) 1,5-pentanediol	0.5m*M* of each Divalent cation II	0.1*M* GlyGly/AMPD pH 8.5
B12	5%(*w*/*v*) PEG 20K, 25%(*w*/*v*) 1,1,1-tris(hydroxymethyl)propane, 1%(*w*/*v*) NDSB 195	0.5m*M* of each Divalent cation II	0.1*M* GlyGly/AMPD pH 8.5
C1	15%(*w*/*v*) PEG 3K, 20%(*v*/*v*) 1,2,4-butanetriol, 1%(*w*/*v*) NDSB 256	1m*M* of each Alkali	0.1*M* MOPSO/bis-tris pH 6.5
C2	12.5%(*w*/*v*) PEG 4K, 20%(*v*/*v*) 1,2,6-hexanetriol	1m*M* of each Alkali	0.1*M* MOPSO/bis-tris pH 6.5
C3	10%(*w*/*v*) PEG 8K, 20%(*v*/*v*) 1,5-pentanediol	1m*M* of each Alkali	0.1*M* MOPSO/bis-tris pH 6.5
C4	5%(*w*/*v*) PEG 20K, 25%(*w*/*v*) 1,1,1-tris(hydroxymethyl)propane, 1%(*w*/*v*) NDSB 195	1m*M* of each Alkali	0.1*M* MOPSO/bis-tris pH 6.5
C5	15%(*w*/*v*) PEG 3K, 20%(*v*/*v*) 1,2,4-butanetriol, 1%(*w*/*v*) NDSB 256	1m*M* of each Alkali	0.1*M* BES/TEA pH 7.5
C6	12.5%(*w*/*v*) PEG 4K, 20%(*v*/*v*) 1,2,6-hexanetriol	1m*M* of each Alkali	0.1*M* BES/TEA pH 7.5
C7	10%(*w*/*v*) PEG 8K, 20%(*v*/*v*) 1,5-pentanediol	1m*M* of each Alkali	0.1*M* BES/TEA pH 7.5
C8	5%(*w*/*v*) PEG 20K, 25%(*w*/*v*) 1,1,1-tris(hydroxymethyl)propane, 1%(*w*/*v*) NDSB 195	1m*M* of each Alkali	0.1*M* BES/TEA pH 7.5
C9	15%(*w*/*v*) PEG 3K, 20%(*v*/*v*) 1,2,4-butanetriol, 1%(*w*/*v*) NDSB 256	1m*M* of each Alkali	0.1*M* GlyGly/AMPD pH 8.5
C10	12.5%(*w*/*v*) PEG 4K, 20%(*v*/*v*) 1,2,6-hexanetriol	1m*M* of each Alkali	0.1*M* GlyGly/AMPD pH 8.5
C11	10%(*w*/*v*) PEG 8K, 20%(*v*/*v*) 1,5-pentanediol	1m*M* of each Alkali	0.1*M* GlyGly/AMPD pH 8.5
C12	5%(*w*/*v*) PEG 20K, 25%(*w*/*v*) 1,1,1-tris(hydroxymethyl)propane, 1%(*w*/*v*) NDSB 195	1m*M* of each Alkali	0.1*M* GlyGly/AMPD pH 8.5
D1	15%(*w*/*v*) PEG 3K, 20%(*v*/*v*) 1,2,4-butanetriol, 1%(*w*/*v*) NDSB 256	0.5m*M* of each Oxometalate	0.1*M* MOPSO/bis-tris pH 6.5
D2	12.5%(*w*/*v*) PEG 4K, 20%(*v*/*v*) 1,2,6-hexanetriol	0.5m*M* of each Oxometalate	0.1*M* MOPSO/bis-tris pH 6.5
D3	10%(*w*/*v*) PEG 8K, 20%(*v*/*v*) 1,5-pentanediol	0.5m*M* of each Oxometalate	0.1*M* MOPSO/bis-tris pH 6.5
D4	5%(*w*/*v*) PEG 20K, 25%(*w*/*v*) 1,1,1-tris(hydroxymethyl)propane, 1%(*w*/*v*) NDSB 195	0.5m*M* of each Oxometalate	0.1*M* MOPSO/bis-tris pH 6.5
D5	15%(*w*/*v*) PEG 3K, 20%(*v*/*v*) 1,2,4-butanetriol, 1%(*w*/*v*) NDSB 256	0.5m*M* of each Oxometalate	0.1*M* BES/TEA pH 7.5
D6	12.5%(*w*/*v*) PEG 4K, 20%(*v*/*v*) 1,2,6-hexanetriol	0.5m*M* of each Oxometalate	0.1*M* BES/TEA pH 7.5
D7	10%(*w*/*v*) PEG 8K, 20%(*v*/*v*) 1,5-pentanediol	0.5m*M* of each Oxometalate	0.1*M* BES/TEA pH 7.5
D8	5%(*w*/*v*) PEG 20K, 25%(*w*/*v*) 1,1,1-tris(hydroxymethyl)propane, 1%(*w*/*v*) NDSB 195	0.5m*M* of each Oxometalate	0.1*M* BES/TEA pH 7.5
D9	15%(*w*/*v*) PEG 3K, 20%(*v*/*v*) 1,2,4-butanetriol, 1%(*w*/*v*) NDSB 256	0.5m*M* of each Oxometalate	0.1*M* GlyGly/AMPD pH 8.5
D10	12.5%(*w*/*v*) PEG 4K, 20%(*v*/*v*) 1,2,6-hexanetriol	0.5m*M* of each Oxometalate	0.1*M* GlyGly/AMPD pH 8.5
D11	10%(*w*/*v*) PEG 8K, 20%(*v*/*v*) 1,5-pentanediol	0.5m*M* of each Oxometalate	0.1*M* GlyGly/AMPD pH 8.5
D12	5%(*w*/*v*) PEG 20K, 25%(*w*/*v*) 1,1,1-tris(hydroxymethyl)propane, 1%(*w*/*v*) NDSB 195	0.5m*M* of each Oxometalate	0.1*M* GlyGly/AMPD pH 8.5
E1	15%(*w*/*v*) PEG 3K, 20%(*v*/*v*) 1,2,4-butanetriol, 1%(*w*/*v*) NDSB 256	0.5m*M* of each Lanthanide	0.1*M* MOPSO/bis-tris pH 6.5
E2	12.5%(*w*/*v*) PEG 4K, 20%(*v*/*v*) 1,2,6-hexanetriol	0.5m*M* of each Lanthanide	0.1*M* MOPSO/bis-tris pH 6.5
E3	10%(*w*/*v*) PEG 8K, 20%(*v*/*v*) 1,5-pentanediol	0.5m*M* of each Lanthanide	0.1*M* MOPSO/bis-tris pH 6.5
E4	5%(*w*/*v*) PEG 20K, 25%(*w*/*v*) 1,1,1-tris(hydroxymethyl)propane, 1%(*w*/*v*) NDSB 195	0.5m*M* of each Lanthanide	0.1*M* MOPSO/bis-tris pH 6.5
E5	15%(*w*/*v*) PEG 3K, 20%(*v*/*v*) 1,2,4-butanetriol, 1%(*w*/*v*) NDSB 256	0.5m*M* of each Lanthanide	0.1*M* BES/TEA pH 7.5
E6	12.5%(*w*/*v*) PEG 4K, 20%(*v*/*v*) 1,2,6-hexanetriol	0.5m*M* of each Lanthanide	0.1*M* BES/TEA pH 7.5
E7	10%(*w*/*v*) PEG 8K, 20%(*v*/*v*) 1,5-pentanediol	0.5m*M* of each Lanthanide	0.1*M* BES/TEA pH 7.5
E8	5%(*w*/*v*) PEG 20K, 25%(*w*/*v*) 1,1,1-tris(hydroxymethyl)propane, 1%(*w*/*v*) NDSB 195	0.5m*M* of each Lanthanide	0.1*M* BES/TEA pH 7.5
E9	15%(*w*/*v*) PEG 3K, 20%(*v*/*v*) 1,2,4-butanetriol, 1%(*w*/*v*) NDSB 256	0.5m*M* of each Lanthanide	0.1*M* GlyGly/AMPD pH 8.5
E10	12.5%(*w*/*v*) PEG 4K, 20%(*v*/*v*) 1,2,6-hexanetriol	0.5m*M* of each Lanthanide	0.1*M* GlyGly/AMPD pH 8.5
E11	10%(*w*/*v*) PEG 8K, 20%(*v*/*v*) 1,5-pentanediol	0.5m*M* of each Lanthanide	0.1*M* GlyGly/AMPD pH 8.5
E12	5%(*w*/*v*) PEG 20K, 25%(*w*/*v*) 1,1,1-tris(hydroxymethyl)propane, 1%(*w*/*v*) NDSB 195	0.5m*M* of each Lanthanide	0.1*M* GlyGly/AMPD pH 8.5
F1	15%(*w*/*v*) PEG 3K, 20%(*v*/*v*) 1,2,4-butanetriol, 1%(*w*/*v*) NDSB 256	0.02*M* of each Monosaccharide II	0.1*M* MOPSO/bis-tris pH 6.5
F2	12.5%(*w*/*v*) PEG 4K, 20%(*v*/*v*) 1,2,6-hexanetriol	0.02*M* of each Monosaccharide II	0.1*M* MOPSO/bis-tris pH 6.5
F3	10%(*w*/*v*) PEG 8K, 20%(*v*/*v*) 1,5-pentanediol	0.02*M* of each Monosaccharide II	0.1*M* MOPSO/bis-tris pH 6.5
F4	5%(*w*/*v*) PEG 20K, 25%(*w*/*v*) 1,1,1-tris(hydroxymethyl)propane, 1%(*w*/*v*) NDSB 195	0.02*M* of each Monosaccharide II	0.1*M* MOPSO/bis-tris pH 6.5
F5	15%(*w*/*v*) PEG 3K, 20%(*v*/*v*) 1,2,4-butanetriol, 1%(*w*/*v*) NDSB 256	0.02*M* of each Monosaccharide II	0.1*M* BES/TEA pH 7.5
F6	12.5%(*w*/*v*) PEG 4K, 20%(*v*/*v*) 1,2,6-hexanetriol	0.02*M* of each Monosaccharide II	0.1*M* BES/TEA pH 7.5
F7	10%(*w*/*v*) PEG 8K, 20%(*v*/*v*) 1,5-pentanediol	0.02*M* of each Monosaccharide II	0.1*M* BES/TEA pH 7.5
F8	5%(*w*/*v*) PEG 20K, 25%(*w*/*v*) 1,1,1-tris(hydroxymethyl)propane, 1%(*w*/*v*) NDSB 195	0.02*M* of each Monosaccharide II	0.1*M* BES/TEA pH 7.5
F9	15%(*w*/*v*) PEG 3K, 20%(*v*/*v*) 1,2,4-butanetriol, 1%(*w*/*v*) NDSB 256	0.02*M* of each Monosaccharide II	0.1*M* GlyGly/AMPD pH 8.5
F10	12.5%(*w*/*v*) PEG 4K, 20%(*v*/*v*) 1,2,6-hexanetriol	0.02*M* of each Monosaccharide II	0.1*M* GlyGly/AMPD pH 8.5
F11	10%(*w*/*v*) PEG 8K, 20%(*v*/*v*) 1,5-pentanediol	0.02*M* of each Monosaccharide II	0.1*M* GlyGly/AMPD pH 8.5
F12	5%(*w*/*v*) PEG 20K, 25%(*w*/*v*) 1,1,1-tris(hydroxymethyl)propane, 1%(*w*/*v*) NDSB 195	0.02*M* of each Monosaccharide II	0.1*M* GlyGly/AMPD pH 8.5
G1	15%(*w*/*v*) PEG 3K, 20%(*v*/*v*) 1,2,4-butanetriol, 1%(*w*/*v*) NDSB 256	0.02*M* of each Amino-acid II	0.1*M* MOPSO/bis-tris pH 6.5
G2	12.5%(*w*/*v*) PEG 4K, 20%(*v*/*v*) 1,2,6-hexanetriol	0.02*M* of each Amino-acid II	0.1*M* MOPSO/bis-tris pH 6.5
G3	10%(*w*/*v*) PEG 8K, 20%(*v*/*v*) 1,5-pentanediol	0.02*M* of each Amino-acid II	0.1*M* MOPSO/bis-tris pH 6.5
G4	5%(*w*/*v*) PEG 20K, 25%(*w*/*v*) 1,1,1-tris(hydroxymethyl)propane, 1%(*w*/*v*) NDSB 195	0.02*M* of each Amino-acid II	0.1*M* MOPSO/bis-tris pH 6.5
G5	15%(*w*/*v*) PEG 3K, 20%(*v*/*v*) 1,2,4-butanetriol, 1%(*w*/*v*) NDSB 256	0.02*M* of each Amino-acid II	0.1*M* BES/TEA pH 7.5
G6	12.5%(*w*/*v*) PEG 4K, 20%(*v*/*v*) 1,2,6-hexanetriol	0.02*M* of each Amino-acid II	0.1*M* BES/TEA pH 7.5
G7	10%(*w*/*v*) PEG 8K, 20%(*v*/*v*) 1,5-pentanediol	0.02*M* of each Amino-acid II	0.1*M* BES/TEA pH 7.5
G8	5%(*w*/*v*) PEG 20K, 25%(*w*/*v*) 1,1,1-tris(hydroxymethyl)propane, 1%(*w*/*v*) NDSB 195	0.02*M* of each Amino-acid II	0.1*M* BES/TEA pH 7.5
G9	15%(*w*/*v*) PEG 3K, 20%(*v*/*v*) 1,2,4-butanetriol, 1%(*w*/*v*) NDSB 256	0.02*M* of each Amino-acid II	0.1*M* GlyGly/AMPD pH 8.5
G10	12.5%(*w*/*v*) PEG 4K, 20%(*v*/*v*) 1,2,6-hexanetriol	0.02*M* of each Amino-acid II	0.1*M* GlyGly/AMPD pH 8.5
G11	10%(*w*/*v*) PEG 8K, 20%(*v*/*v*) 1,5-pentanediol	0.02*M* of each Amino-acid II	0.1*M* GlyGly/AMPD pH 8.5
G12	5%(*w*/*v*) PEG 20K, 25%(*w*/*v*) 1,1,1-tris(hydroxymethyl)propane, 1%(*w*/*v*) NDSB 195	0.02*M* of each Amino-acid II	0.1*M* GlyGly/AMPD pH 8.5
H1	15%(*w*/*v*) PEG 3K, 20%(*v*/*v*) 1,2,4-butanetriol, 1%(*w*/*v*) NDSB 256	0.01*M* of each Polyamine	0.1*M* MOPSO/bis-tris pH 6.5
H2	12.5%(*w*/*v*) PEG 4K, 20%(*v*/*v*) 1,2,6-hexanetriol	0.01*M* of each Polyamine	0.1*M* MOPSO/bis-tris pH 6.5
H3	10%(*w*/*v*) PEG 8K, 20%(*v*/*v*) 1,5-pentanediol	0.01*M* of each Polyamine	0.1*M* MOPSO/bis-tris pH 6.5
H4	5%(*w*/*v*) PEG 20K, 25%(*w*/*v*) 1,1,1-tris(hydroxymethyl)propane, 1%(*w*/*v*) NDSB 195	0.01*M* of each Polyamine	0.1*M* MOPSO/bis-tris pH 6.5
H5	15%(*w*/*v*) PEG 3K, 20%(*v*/*v*) 1,2,4-butanetriol, 1%(*w*/*v*) NDSB 256	0.01*M* of each Polyamine	0.1*M* BES/TEA pH 7.5
H6	12.5%(*w*/*v*) PEG 4K, 20%(*v*/*v*) 1,2,6-hexanetriol	0.01*M* of each Polyamine	0.1*M* BES/TEA pH 7.5
H7	10%(*w*/*v*) PEG 8K, 20%(*v*/*v*) 1,5-pentanediol	0.01*M* of each Polyamine	0.1*M* BES/TEA pH 7.5
H8	5%(*w*/*v*) PEG 20K, 25%(*w*/*v*) 1,1,1-tris(hydroxymethyl)propane, 1%(*w*/*v*) NDSB 195	0.01*M* of each Polyamine	0.1*M* BES/TEA pH 7.5
H9	15%(*w*/*v*) PEG 3K, 20%(*v*/*v*) 1,2,4-butanetriol, 1%(*w*/*v*) NDSB 256	0.01*M* of each Polyamine	0.1*M* GlyGly/AMPD pH 8.5
H10	12.5%(*w*/*v*) PEG 4K, 20%(*v*/*v*) 1,2,6-hexanetriol	0.01*M* of each Polyamine	0.1*M* GlyGly/AMPD pH 8.5
H11	10%(*w*/*v*) PEG 8K, 20%(*v*/*v*) 1,5-pentanediol	0.01*M* of each Polyamine	0.1*M* GlyGly/AMPD pH 8.5
H12	5%(*w*/*v*) PEG 20K, 25%(*w*/*v*) 1,1,1-tris(hydroxymethyl)propane, 1%(*w*/*v*) NDSB 195	0.01*M* of each Polyamine	0.1*M* GlyGly/AMPD pH 8.5

**Table 2:** Selection of the 35 PDB-derived ligands selected to formulate MORPHEUS II Number of structures (*i.e.* occurrences in the PDB) showing a main chemical as an ordered ligand (as of December 2014). Note that a few reagents that were not found in the PDB were integrated to complete the formulations (the four polyols used as cryoprotectants and five of the six buffers used to formulate the buffer systems).

Chemical name	Type	PDB ID (main)	No. of structures
Lithium sulfate	Common salt	LI	51
Sodium chloride	Common salt	NA	4726
Potassium sulfate	Common salt	K	1638
Manganese chloride tetrahydrate	Divalent cation	MN	1938
Cobalt chloride hexahydrate	Divalent cation	CO	474
Nickel chloride hexahydrate	Divalent cation	NI	699
Zinc acetate dihydrate	Divalent cation	ZN	8413
Barium acetate	Alkali	BA	91
Cesium acetate	Alkali	CS	75
Rubidium chloride	Alkali	RB	34
Strontium acetate	Alkali	SR	101
Sodium chromate tetrahydrate	Oxometalate	CR	7
Sodium molybdate dihydrate	Oxometalate	MOO	20
Sodium orthovanadate	Oxometalate	VO4	73
Sodium tungstate dihydrate	Oxometalate	WO4	47
Erbium(III) chloride hexahydrate	Lanthanide	ER3	2
Terbium(III) chloride hexahydrate	Lanthanide	TB	11
Ytterbium(III) chloride hexahydrate	Lanthanide	YB	57
Yttrium(III) chloride hexahydrate	Lanthanide	YT3	33
Xylitol	Monosaccharide	XYL	25
D-()-Fructose	Monosaccharide	FRU; FUD	36; 4
D-Sorbitol	Monosaccharide	SOR	12
*myo*-Inositol	Monosaccharide	INS	16
L-Rhamnose monohydrate	Monosaccharide	RAM	43
DL-Threonine	Amino-acid	DTH; THR	23; n/a
DL-HistidineHClH_2_O	Amino-acid	DHI; HIS	24; n/a
DL-5-HydroxylysineHCl	Amino-acid	n/a; LYZ	0; 7
*trans*-4-Hydroxy-L-proline	Amino-acid	HYP	149
Spermine4HCl	Polyamine	SPM	105
Spermidine3HCl	Polyamine	SPD	33
1,4-Diaminobutane2HCl	Polyamine	PUT	22
DL-OrnithineHCl	Polyamine	ORD; ORN	3; 56
NDSB 256	Surfactant	DMX	4
NDSB 195	Surfactant	NDS	7
Bis-tris	Buffer	BTB	114

**Table 3:** Formulation of the eight additive mixes of MORPHEUS II Note that the mix called Alkalis includes two of the alkali-earth metals, strontium and barium, and two of the alkali metals, cesium and rubidium. Also, the mix called Lanthanides includes one of the rare-earth elements chemically very similar to the lanthanides (yttrium).

Row	Mix name	Chemicals
A	LiNaK	0.3*M* lithium sulfate, 0.3*M* sodium sulfate, 0.3*M* potassium sulfate
B	Divalent cations II	5m*M* manganese chloride, 5m*M* cobalt chloride, 5m*M* nickel chloride, 5m*M* zinc chloride
C	Alkalis	10m*M* rubidium chloride, 10m*M* strontium acetate, 10m*M* cesium acetate, 10m*M* barium acetate
D	Oxometalates	5m*M* sodium chromate, 5m*M* sodium molybdate, 5m*M* sodium tungstate, 5m*M* sodium orthovanadate
E	Lanthanides	5m*M* erbium(III) chloride hexahydrate, 5m*M* terbium(III) chloride hexahydrate, 5m*M* ytterbium(III) chloride hexahydrate, 5m*M* yttrium(III) chloride hexahydrate
F	Monosaccharides II	0.2*M* xylitol, 0.2*M* D-()-fructose, 0.2*M* D-sorbitol, 0.2*M* *myo*-inositol, 0.2*M* L-rhamnose monohydrate
G	Amino-acids II	0.2*M* DL-arginineHCl, 0.2*M* DL-threonine, 0.2*M* DL-histidineHClH_2_O, 0.2*M* DL-5-hydroxylysineHCl, 0.2*M* *trans*-4-hydroxy-L-proline
H	Polyamines	0.1*M* spermine4HCl, 0.1*M* spermidine3HCl, 0.1*M* 1,4-diaminobutane2HCl, 0.1*M* DL-ornithineHCl

## References

[bb1] Abola, E., Kuhn, P., Earnest, T. & Stevens, R. C. (2000). *Nature Struct. Biol.* **7**, 973–977. 10.1038/80754 11104004

[bb2] Aibara, S., Katahira, J., Valkov, E. & Stewart, M. (2015). *Nucleic Acids Res.* **43**, 1883–1893. 10.1093/nar/gkv032 PMC4330390 25628361

[bb3] Alcorn, T. & Juers, D. H. (2010). *Acta Cryst.* D**66**, 366–373. 10.1107/S090744490903995X PMC2852300 20382989

[bb5] Arakawa, T. & Timasheff, S. N. (1982). *Biochemistry*, **21**, 6536–6544. 10.1021/bi00268a033 7150574

[bb6] Berejnov, V., Husseini, N. S., Alsaied, O. A. & Thorne, R. E. (2006). *J. Appl. Cryst.* **39**, 244–251. 10.1107/S0021889806037484 PMC2866519 20461232

[bb7] Bergfors, T. M. (2009). *Protein Crystallization*, 2nd ed. La Jolla: International University Line.

[bb8] Birtley, J. R., Saridakis, E., Stratikos, E. & Mavridis, I. M. (2012). *Biochemistry*, **51**, 286–295. 10.1021/bi201230p 22106953

[bb9] Blow, D. (2002). *Outline of Crystallography for Biologists.* Oxford University Press.

[bb10] Boggon, T. J. & Shapiro, L. (2000). *Structure*, **8**, 143–149. 10.1016/s0969-2126(00)00168-4 10903954

[bb11] Bolton, P. H. & Kearns, D. R. (1978). *Nucleic Acids Res.* **5**, 1315–1324. 10.1093/nar/5.4.1315 PMC342079 652523

[bb12] Carter, C. W. & Carter, C. W. (1979). *J. Biol. Chem.* **254**, 12219–12223. 500706

[bb13] Carugo, O. & Argos, P. (1997). *Protein Sci.* **6**, 2261–2263. 10.1002/pro.5560061021 PMC2143556 9336849

[bb14] Carugo, O. & Djinović-Carugo, K. (2014). *J. Appl. Cryst.* **47**, 458–461.

[bb15] Cohen, S., Marcus, Y., Migron, Y., Dikstein, S. & Shafran, S. (1993). *Faraday Trans.* **89**, 3271–3275.

[bb16] Collins, K. D. (2004). *Methods*, **34**, 300–311. 10.1016/j.ymeth.2004.03.021 15325648

[bb17] Danley, D. E. (2006). *Acta Cryst.* D**62**, 569–575. 10.1107/S0907444906012601 16699182

[bb18] Drew, H. R. & Dickerson, R. E. (1981). *J. Mol. Biol.* **151**, 535–556. 10.1016/0022-2836(81)90009-7 7338904

[bb19] Elliott, P. R., Nielsen, S. V., Marco-Casanova, P., Fiil, B. K., Keusekotten, K., Mailand, N., Freund, S. M., Gyrd-Hansen, M. & Komander, D. (2014). *Mol. Cell*, **54**, 335–348. 10.1016/j.molcel.2014.03.018 PMC4017264 24726323

[bb20] Ericsson, U. B., Hallberg, B. M., DeTitta, G. T., Dekker, N. & Nordlund, P. (2006). *Anal. Biochem.* **357**, 289–298. 10.1016/j.ab.2006.07.027 16962548

[bb21] Fazio, V. J., Peat, T. S. & Newman, J. (2014). *Acta Cryst.* F**70**, 1303–1311. 10.1107/S2053230X1401841X PMC4188070 25286930

[bb22] Fersht, A. R. (2008). *Nature Rev. Mol. Cell Biol.* **9**, 650–654. 10.1038/nrm2446 18578032

[bb23] Forsythe, E. L., Maxwell, D. L. & Pusey, M. (2002). *Acta Cryst.* D**58**, 1601–1605. 10.1107/s0907444902014208 12351870

[bb24] Garman, E. F. & Mitchell, E. P. (1996). *J. Appl. Cryst.* **29**, 584–587.

[bb25] Garman, E. & Murray, J. W. (2003). *Acta Cryst.* D**59**, 1903–1913. 10.1107/s0907444903012794 14573944

[bb26] Gorrec, F. (2009). *J. Appl. Cryst.* **42**, 1035–1042. 10.1107/S0021889809042022 PMC3246824 22477774

[bb27] Gorrec, F. (2013). *J. Appl. Cryst.* **46**, 795–797. 10.1107/S0021889813008030 PMC3654315 23682195

[bb28] Gorrec, F. (2014). *Drug Discov. Today*, **19**, 1505–1507. 10.1016/j.drudis.2014.07.002 PMC4874463 25048082

[bb29] Izaac, A., Schall, C. A. & Mueser, T. C. (2006). *Acta Cryst.* D**62**, 833–842. 10.1107/S0907444906018385 16790940

[bb30] Jancarik, J. & Kim, S.-H. (1991). *J. Appl. Cryst.* **24**, 409–411.

[bb71] Kantardjieff, K. A. & Rupp, B. (2004). *Bioinformatics*, **20**, 2162–2168. 10.1093/bioinformatics/bth066 14871873

[bb31] Khurshid, S., Saridakis, E., Govada, L. & Chayen, N. E. (2014). *Nature Protoc.* **9**, 1621–1633. 10.1038/nprot.2014.109 24922271

[bb32] Kimber, M. S., Vallee, F., Houston, S., Nečakov, A., Skarina, T., Evdokimova, E., Beasley, S., Christendat, D., Savchenko, A., Arrowsmith, C. H., Vedadi, M., Gerstein, M. & Edwards, A. M. (2003). *Proteins*, **51**, 562–568. 10.1002/prot.10340 12784215

[bb33] Lomakin, A., Asherie, N. & Benedek, G. B. (1999). *Proc. Natl Acad. Sci. USA*, **96**, 9465–9468. 10.1073/pnas.96.17.9465 PMC22231 10449715

[bb34] Luft, J. R., Newman, J. & Snell, E. H. (2014). *Acta Cryst.* F**70**, 835–853. 10.1107/S2053230X1401262X PMC4089519 25005076

[bb35] Maclean, D. S., Qian, Q. & Middaugh, C. R. (2002). *J. Pharm. Sci.* **91**, 2220–2229. 10.1002/jps.10219 12226849

[bb36] Majeed, S., Ofek, G., Belachew, A., Huang, C.-C., Zhou, T. & Kwong, P. D. (2003). *Structure*, **11**, 1061–1070. 10.1016/s0969-2126(03)00185-0 12962625

[bb37] McFerrin, M. B. & Snell, E. H. (2002). *J. Appl. Cryst.* **35**, 538–545.

[bb38] McPherson, A. & Cudney, B. (2006). *J. Struct. Biol.* **156**, 387–406. 10.1016/j.jsb.2006.09.006 17101277

[bb39] McPherson, A., Malkin, A. J. & Kuznetsov, Y. G. (1995). *Structure*, **3**, 759–768. 10.1016/s0969-2126(01)00211-8 7582893

[bb40] Newman, J. (2004). *Acta Cryst.* D**60**, 610–612. 10.1107/S0907444903029640 14993709

[bb55] Newman, J., Egan, D., Walter, T. S., Meged, R., Berry, I., Ben Jelloul, M., Sussman, J. L., Stuart, D. I. & Perrakis, A. (2005). *Acta Cryst.* D**61**, 1426–1431. 10.1107/S0907444905024984 16204897

[bb42] Newstead, S., Ferrandon, S. & Iwata, S. (2008). *Protein Sci.* **17**, 466–472. 10.1110/ps.073263108 PMC2248303 18218713

[bb41] Nguyen, J. B. & Modis, Y. (2013). *J. Struct. Biol.* **181**, 82–88. 10.1016/j.jsb.2012.10.006 PMC3525766 23099021

[bb43] Niemann, V., Koch-Singenstreu, M., Neu, A., Nilkens, S., Götz, F., Unden, G. & Stehle, T. (2014). *J. Mol. Biol.* **426**, 1539–1553. 10.1016/j.jmb.2013.12.026 24389349

[bb44] Page, R. & Stevens, R. C. (2004). *Methods*, **34**, 373–389. 10.1016/j.ymeth.2004.03.026 15325655

[bb45] Peter, B. J., Kent, H. M., Mills, I. G., Vallis, Y., Butler, P. J., Evans, P. R. & McMahon, H. T. (2004). *Science*, **303**, 495–499. 10.1126/science.1092586 14645856

[bb46] Petsko, G. A. (1975). *J. Mol. Biol.* **96**, 381–392. 10.1016/0022-2836(75)90167-9 240944

[bb47] Petsko, G. A. (1985). *Methods Enzymol.* **114**, 147–156. 10.1016/0076-6879(85)14015-2 4079763

[bb48] Price, A. J., Jacques, D. A., McEwan, W. A., Fletcher, A. J., Essig, S., Chin, J. W., Halambage, U. D., Aiken, C. & James, L. C. (2014). *PLoS Pathog.* **10**, e1004459. 10.1371/journal.ppat.1004459 PMC4214760 25356722

[bb49] Pusey, M. L., Paley, M. S., Turner, M. B. & Rogers, R. D. (2007). *Cryst. Growth Des.* **7**, 787–793.

[bb50] Rupp, B. (2003). *J. Struct. Biol.* **142**, 162–169. 10.1016/s1047-8477(03)00047-9 12718928

[bb51] Rupp, B. (2010). *Biomolecular Crystallography: Principles, Practice and Applications to Structural Biology*. New York: Garland Science.

[bb52] Rupp, B. & Wang, J. (2004). *Methods*, **34**, 390–407. 10.1016/j.ymeth.2004.03.031 15325656

[bb53] Sauter, C., Ng, J. D., Lorber, B., Keith, G., Brion, P., Hosseini, M. W., Lehn, J.-M. & Giegé, R. (1999). *J. Cryst. Growth*, **196**, 365–376.

[bb54] Stock, D., Perisic, O. & Löwe, J. (2005). *Prog. Biophys. Mol. Biol.* **88**, 311–327. 10.1016/j.pbiomolbio.2004.07.009 15652247

[bb56] Taylor, G. (2003). *Acta Cryst.* D**59**, 1881–1890. 10.1107/s0907444903017815 14573942

[bb57] Teo, H., Perisic, O., González, B. & Williams, R. L. (2004). *Dev. Cell*, **7**, 559–569. 10.1016/j.devcel.2004.09.003 15469844

[bb58] Toste Rêgo, A., Holding, A. N., Kent, H. & Lamers, M. H. (2013). *EMBO J.* **32**, 1334–1343. 10.1038/emboj.2013.68 PMC3642679 23549287

[bb59] Trakhanov, S. & Quiocho, F. A. (1995). *Protein Sci.* **4**, 1914–1919. 10.1002/pro.5560040925 PMC2143217 8528088

[bb60] Vuillard, L., Baalbaki, B., Lehmann, M., Nørager, S., Legrand, P. & Roth, M. (1996). *J. Cryst. Growth*, **168**, 150–154.

[bb61] Wooh, J. W., Kidd, R. D., Martin, J. L. & Kobe, B. (2003). *Acta Cryst.* D**59**, 769–772. 10.1107/s0907444903002919 12657807

[bb62] Zhang, F., Zocher, G., Sauter, A., Stehle, T. & Schreiber, F. (2011). *J. Appl. Cryst.* **44**, 755–762.

