# Supplementary material for: The MORPHEUS II protein crystallization screen
Source: Acta Crystallogr F Struct Biol Commun. 2015 Jun 27;71(Pt 7):831–7. doi: 10.1107/S2053230X1500967X (PMC4498703; doi:10.1107/S2053230X1500967X)
Supplement: Supplementary file 1 [file f-71-00831-sup1.pdf]

Acta Cryst F

STRUCTURAL BIOLOGY COMMUNICATIONS

Volume 71 (2015)

Supporting information for article:

The MORPHEUS II protein crystallization screen

Fabrice Gorrec

Table S1 MORPHEUS II precipitant mixes.

Wells	Prec.	Conc	Unit	Cryo	Conc.	Unit	Surfactant	Conc.	Unit
Col 1, 5 and 9	PEG 3K	30	% w/v	1,2,4-Butanetriol	40	% v/v	NDSB 256	2	% w/v
Col 2, 6 and 10	PEG 4K	25	% w/v	1,2,6-Hexanetriol	40	% v/v			
Col 3, 7 and 11	PEG 8K	20	% w/v	1,5-Pentanediol	40	% v/v			
Col 4, 8 and 12	PEG 20K	10	% w/v	1,1,1-Tris(hydroxymethyl)propane	50	% w/v	NDSB 195	2	% w/v

Table S2 MORPHEUS II buffer systems.

Wells	Acid name	Conc	Unit	pH	Base name	Conc	Unit	pH	ratio (approx.)	final pH
Col 1-4	MOPSO	1	M	3.3	Bis-tris	1	M	12.0	60/40	6.5
Col 5-8	BES	1	M	4.16	Triethylamine (TEA)	1	M	11.2	50/50	7.5
Col 9-12	GlyGly	1	M	5.8	AMPD	1	M	11.8	66/33	8.5

Table S3 Crystallisation results

A condition was considered as succesful when at least one of the triplicate exhibited crystals (results were all repeated at least twice apart from rare exceptions). The yield is calculated as follow: Total ÷ (96 × 8) × 100.

JCSG+

Well	Con.	Pol.	E2H	Bar	CCD	UPL	NEF	HIV
A1								
A2	x			x				
A3	x					x		
A4								
A5								
A6		x					x	
A7	x							
A8	x				x			x
A9	x			x				x
A10	x				x	x		
A11								
A12	x			x	x	x		x
B1								
B2				x	x	x		
B3								
B4					x			x
B5					x			
B6								
B7		x						x
B8	x		x				x	
B9	x	x						
B10								
B11								
B12				x				
C1		x						
C2							x	
C3	x			x				x
C4	x							x
C5								
C6								
C7								
C8								
C9					x			
C10								
C11								
C12		x						x
D1								
D2								x
D3								
D4								
D5								
D6	x							
D7								
D8								
D9								
D10		x						
D11								
D12	x							

MORPHEUS I

Well	Con.	Pol.	E2H	Bar	CCD	UPL	NEF	HIV
A1	x	x						
A2								x
A3								
A4		x						
A5		x						
A6				x	x			
A7								
A8		x						
A9		x						
A10						x		
A11						x	x	
A12		x						
B1	x	x						
B2	x							x
B3								
B4		x						
B5		x			x			
B6								
B7					x	x		
B8		x			x			
B9		x						x
B10								x
B11						x		x
B12		x			x			
C1	x	x						x
C2					x			x
C3								x
C4		x		x	x			
C5		x						
C6								
C7								
C8		x		x	x			
C9								
C10								x
C11								x
C12		x						x
D1	x	x						
D2	x							x
D3	x							x
D4	x	x						
D5		x			x			
D6								x
D7								x
D8		x						
D9								
D10							x	x
D11						x	x	x
D12		x			x			

MORPHEUS II

Well	Con.	Pol.	E2H	Bar	CCD	UPL	NEF	HIV
A1					x			x
A2	x				x		x	x
A3	x				x			x
A4					x			
A5	x							x
A6	x						x	x
A7	x							x
A8								
A9						x		x
A10	x						x	x
A11	x		x					x
A12			x					
B1	x							
B2	x				x			
B3	x				x			
B4								
B5	x							
B6	x							
B7	x				x			
B8								
B9	x					x		x
B10								x
B11								
B12								
C1	x							
C2	x							x
C3	x				x			x
C4					x			
C5	x							x
C6								x
C7							x	x
C8								
C9	x				x	x		x
C10								x
C11								x
C12								
D1	x							
D2	x				x			
D3	x				x		x	
D4					x			
D5							x	x
D6								
D7								
D8								
D9	x					x		x
D10								x
D11							x	
D12								

JCSG+

Well	Con.	Pol.	E2H	Bar	CCD	UPL	NEF	HIV
**E1**								
**E2**							x	
**E3**								
**E4**					x		x	
**E5**								
**E6**	x							
**E7**								
**E8**								
**E9**							x	
**E10**								
**E11**				x				
**E12**								x
**F1**								
**F2**								
**F3**								
**F4**								
**F5**								
**F6**								
**F7**							x	
**F8**								
**F9**								
**F10**								
**F11**								
**F12**								
**G1**								
**G2**					x	x		
**G3**								
**G4**								x
**G5**	x							
**G6**	x					x		
**G7**	x							x
**G8**	x				x	x		
**G9**								x
**G10**		x						x
**G11**		x						
**G12**								
**H1**								
**H2**								
**H3**		x						
**H4**								
**H5**	x							
**H6**	x			x				x
**H7**		x						
**H8**		x						x
**H9**		x						
**H10**	x	x						x
**H11**	x	x						
**H12**	x							
**Sum**	23	14	1	8	10	7	7	17
						**Total**		87
						**Yield (%)**		11.3

MORPHEUS I

Well	Con.	Pol.	E2H	Bar	CCD	UPL	NEF	HIV
**E1**	x	x						
**E2**	x							x
**E3**	x							x
**E4**	x	x						
**E5**		x						
**E6**								
**E7**								x
**E8**		x						
**E9**		x						
**E10**								x
**E11**						x		x
**E12**		x			x			
**F1**		x						
**F2**								
**F3**								
**F4**		x						
**F5**		x						
**F6**								x
**F7**								
**F8**		x						
**F9**								
**F10**								x
**F11**						x		x
**F12**		x			x			
**G1**	x	x						
**G2**								
**G3**								
**G4**	x	x						
**G5**		x						
**G6**								
**G7**								
**G8**		x		x				
**G9**		x						
**G10**							x	x
**G11**						x	x	x
**G12**		x		x				
**H1**	x	x						x
**H2**	x							x
**H3**								x
**H4**	x	x						
**H5**	x	x					x	
**H6**								x
**H7**								x
**H8**		x						
**H9**		x						
**H10**					x			x
**H11**					x	x		x
**H12**		x			x			
	18	45	0	5	15	9	6	34
						**Total**		132
						**Yield (%)**		17.2

MORPHEUS II

Well	Con.	Pol.	E2H	Bar	CCD	UPL	NEF	HIV
**E1**		x						
**E2**		x						
**E3**		x						
**E4**					x			
**E5**	x							x
**E6**								
**E7**								
**E8**								
**E9**	x				x	x		x
**E10**					x			x
**E11**					x		x	
**E12**								
**F1**	x							
**F2**								x
**F3**					x			
**F4**					x			
**F5**	x							x
**F6**								
**F7**								x
**F8**								
**F9**	x				x	x		x
**F10**					x			x
**F11**							x	
**F12**								
**G1**	x							x
**G2**	x							x
**G3**	x						x	x
**G4**					x			
**G5**	x							
**G6**					x			
**G7**					x		x	x
**G8**								
**G9**						x		x
**G10**					x			x
**G11**					x		x	x
**G12**								
**H1**		x						
**H2**								
**H3**							x	
**H4**					x			
**H5**				x		x		
**H6**								
**H7**								
**H8**								
**H9**				x		x		
**H10**								
**H11**					x			
**H12**								
	32	4	2	2	28	9	13	37
						**Total**		127
						**Yield (%)**		16.5
